# Osteogenic and Biocompatibility Potential of Polylactic Acid-Based Materials: A Systematic Review of Human Primary Cells Studies

**DOI:** 10.3390/jfb17010034

**Published:** 2026-01-09

**Authors:** Mario Guerrero-Torres, Silvia M. Becerra-Bayona, Martha L. Arango-Rodríguez, Emilio A. Cafferata

**Affiliations:** 1 Faculty of Dentistry, Universidad Santo Tomas de Aquino, Bucaramanga 680006, Colombia; 2 Facultad de Ciencias de la Salud, Universidad Autónoma de Bucaramanga—UNAB, Bucaramanga 681003, Colombia; 3 Banco Multitejidos y Centro de Terapias Avanzadas, Clínica FOSCAL Internacional, Floridablanca 681004, Colombia; 4 Oral Peri-Implant Research Group, School of Dentistry, Universidad Científica del Sur, Lima 15833, Peru

**Keywords:** polylactic acid, guided bone regeneration, osteoblasts, human primary cells, tissue engineering, biomaterials, fibroblast, bioactivity

## Abstract

Background: Guided Bone Regeneration (GBR) relies on barrier membranes, for which polylactic acid (PLA) and its copolymer poly(lactic-co-glycolic acid) (PLGA) are promising biodegradable polymers. However, their inherent hydrophobicity limits biological performance, and the evidence regarding how specific modifications affect key human cell types, particularly osteoblasts and fibroblasts, remains scattered. Methods: A systematic review was conducted to synthesize the in vitro evidence on the response of primary human osteoblasts and fibroblasts to polylactic acid-based materials. Following a pre-registered protocol (10.17605/OSF.IO/CE8KB), a comprehensive search was performed across four major databases, and the risk of bias in the included studies was assessed using an adapted OHAT tool. Results: Twenty-six studies were included, which showed that polylactic acid-based materials have limited bioactivity, and their modification significantly improves cellular responses. The incorporation of bioceramics and growth factors, or alterations in surface topography, notably enhanced osteogenic differentiation and mineralization in osteoblasts. For gingival fibroblasts, topographical modifications like micro-grooves guided cell alignment and modulated proliferation. Conclusions: Native polylactic acid-based materials display limited bioactivity. However, functionalization through bioceramics incorporation, growth factor delivery, and surface topographical modification is crucial for transforming them into bioactive scaffolds capable of achieving the dual biofunctionality required for successful GBR.

## 1. Introduction

Guided bone regeneration is a cornerstone technique for the management of alveolar bone defects arising from tooth loss or periodontal disease, and implant site development [1]. Its clinical success is critically dependent on the use of a barrier membrane that selectively promotes bone regeneration while preventing the ingrowth of soft tissue. However, currently available membranes present inherent limitations [2].

Non-resorbable membranes, made from expanded polytetrafluoroethylene (e-PTFE) and titanium mesh, provide superior space maintenance and structural integrity but require a second surgical procedure for removal and are highly susceptible to bacterial contamination if exposed [3]. On the other hand, resorbable membranes, typically collagen-based, eliminate the need for membrane retrieval but often lack sufficient mechanical stability, frequently leading to the collapse of the regenerative space, present inconsistent resorption rates and limited bioactivity [3]. These limitations highlight the ongoing demand for a next-generation membrane that combines complete resorbability with the mechanical robustness of its non-resorbable counterparts, a predictable degradation profile, and osteogenic potential. While there is no universal solution for every clinical situation, it is widely acknowledged that the future of GBR lies in the development of membranes capable of promoting bone regeneration, rather than serving as passive barriers [2,3].

Polylactic acid (PLA), a synthetic biodegradable polymer, has emerged as a promising material for GBR resorbable membranes development, due to its favorable biocompatibility, tunable degradation profile and adjustable mechanical properties. It has been investigated for a wide range of clinical applications, including horizontal and vertical ridge augmentation, treatment of intrabony periodontal defects, and coverage of implant-related dehiscence and fenestration defects [1]. Despite its potential, the clinical translation of unmodified PLA remains hampered by intrinsic limitations [4]. First, its inherent hydrophobicity interferes with early biological events, such as blood clot stabilization and protein adsorption, critical for cell adhesion and tissue integration [5]. Then, PLA degradation produces acidic byproducts, primarily lactic acid, which can lower the local microenvironment pH. This acidic shift may not only compromise cell viability but also activate immune cells, such as macrophages, through metabolic reprogramming [6]. This can lead to a sterile inflammatory response characterized by increased proinflammatory cytokines production, which may promote fibrous encapsulation, impair osseous integration and, in some cases, induce localized bone resorption instead of regeneration [7]. These biological drawbacks have driven the development of numerous modifications aimed at transforming PLA from a passive barrier into a bioactive scaffold capable of supporting and promoting bone regeneration [8].

To overcome these limitations, a broad array of physicochemical and biological strategies have been explored to enhance the functionality of PLA [9]. These include the incorporation of bioceramics such as hydroxyapatite (HA), tricalcium phosphate (TCP), amorphous calcium phosphate, or bioactive glass to enhance osteoconductivity [10,11]; blending with biopolymers like collagen to improve cellular affinity and matrix interaction; surface functionalization with inorganic coatings or bioactive molecules to modulate cell behavior; and the incorporation of growth factors such as bone morphogenetic protein (BMP)-2 or transforming growth factor (TGF)-β1 to actively promote osteogenic differentiation and tissue regeneration [9,12,13].

To start, guide and validate the rational development of next-generation PLA membranes, a thorough understanding of their interaction with the primary human cells most relevant to GBR is required. These mainly include osteoblasts, responsible for bone formation, and gingival fibroblasts, which are critical for soft tissue sealing and barrier integration. Therefore, an ideal membrane must exhibit a “dual biofunctionality”: its internal surface (facing the bone defect) should be osteoinductive, while the external surface (facing the soft tissue) should support the integration with the connective tissue and prevent epithelial ingrowth [2].

Despite substantial progress, the current body of in vitro evidence regarding primary human cell responses to PLA membranes and its modifications remains scattered and often based on non-human or immortalized cell models. These models often exhibit altered genetic, phenotypic, and signaling profiles leading to altered responses to biomaterial properties and limited physiological relevance. In contrast, primary human cells retain native characteristics and donor-specific variability, offering a more accurate representation of clinical conditions [14]. This lack of consolidated data hinders the identification of the most effective material modifications and prevents progress towards the design of clinically optimized PLA membranes for GBR [15,16].

In this review, we use the term “PLA-based materials” to encompass both polylactic-acid homopolymers (PLLA/PDLLA) and the copolymer PLGA. This choice is justified because they share the lactic repeat unit and hydrolytic degradation pathway with lactic acid release; PLGA is widely used as a barrier membrane for GBR and undergoes the same families of modifications and comparing PLA vs. PLGA helps disentangle the influence of copolymer composition, crystallinity, and degradation kinetics on primary human osteoblast/progenitor and gingival fibroblast responses.

Therefore, the objective of this systematic review was to address a specific and under-synthesized question: how polylactic acid–based membranes and their modifications influence the behavior of primary human osteoblasts/progenitors and gingival fibroblasts relevant to GBR. Previous reviews have provided valuable overviews of this field, yet they have largely relied on heterogeneous sources of evidence, including observational human studies [17], diverse and non-comparable animal models [18], immortalized cell lines [19], and network meta-analyses that aggregate animal data without discriminating species or defect models [20]. In contrast, the present analysis focuses exclusively on primary human cells to maximize translational relevance. The evidence is interpreted within the framework of the dual biofunctionality requirement for GBR membranes—osteogenic performance towards the defect and soft-tissue integration on the opposite face—allowing material modifications to be evaluated against clear, clinically grounded functions. Beyond a descriptive synthesis, the review employs standardized data extraction by outcome domains and systematically maps categories of PLA-based material modifications (inorganic phase incorporation, surface chemistry and energy tuning, topographical control, and immobilized bioactive cues) to specific cellular responses. This translational and function-oriented approach generates evidence-based design principles that are directly actionable for the development and refinement of next-generation PLA-based GBR membranes.

## 2. Materials and Methods

### 2.1. Protocol and Registration

The protocol for this systematic review was registered a priori in the Open Science Frameworks (OSF) Registries platform (Charlottesville, VA, USA) under the digital object identifier (DOI): 10.17605/OSF.IO/CE8KB. The review was conducted and reported in accordance with the PRISMA 2020 statement, ensuring transparency and reproducibility of the methodology employed [21].

### 2.2. Eligibility Criteria

The present systematic review was designed to answer the following PICO research question:

In primary human osteoblast and/or gingival fibroblast cultures (P), what is the cellular response—including viability, proliferation, adhesion, osteogenic differentiation, extracellular matrix production, and inflammatory response—(O) upon exposure to polylactic acid-based materials and its various modifications (I), compared to negative controls, conventional barrier membranes for GBR, or alternative polylactic acid-based materials formulations (C)?

#### 2.2.1. Inclusion Criteria

- Study type: in vitro studies
- Cell population: studies using primary cultures of human osteoblasts (including those derived from mesenchymal stem cells) or primary cultures of human gingival fibroblasts.
- Intervention: studies evaluating the cellular response to pure polylactic acid-based materials or any of its modifications.
- Comparison: studies that included at least one comparison against a control group, standard barrier membranes used in GBR, or between different polylactic acid-based materials formulations.
- Outcomes: studies that reported at least one outcome of interest, such as cell viability, proliferation, adhesion, osteogenic differentiation markers, mineralization, or extracellular matrix production.

#### 2.2.2. Exclusion Criteria

- Study type: letters to the editor, editorials, conference abstracts, and book chapters.
- Cell population: studies using immortalized cell lines, non-human cells, or infected or genetically modified human cells.
- Accessibility: articles whose full text was not available.

Studies using immortalized cell lines and animal (in vivo) models were excluded to avoid nonspecific responses to PLA material modifications that fall outside the objectives of this review.

### 2.3. Information Sources and Search Strategy

A comprehensive literature search was conducted from inception up to October 2025 in four electronic databases: Medline (via Pubmed), Biblioteca Virtual En Salud (BVS), Scopus, and Web of Science. The search strategies combined controlled vocabulary terms and free-text terms to cover all aspects of the PICO question. Additionally, a manual search of the reference lists of the included studies was performed to identify relevant records not captured in the initial search. No date or language restrictions were applied.

### 2.4. Selection of Studies

The selection process was carried out in three phases. First, duplicates were removed using Rayyan software (Version 1.7, Cambridge, MA, USA). Next, two independent reviewers screened the titles and abstracts. Finally, the same two reviewers independently assessed the full text of the pre-selected articles to determine their final eligibility based on the inclusion and exclusion criteria. Any discrepancies during the process were resolved through discussion or with the intervention of a third reviewer to reach a consensus.

### 2.5. Data Collection

Data extraction was performed by two authors independently and in duplicate, using a standardized spreadsheet. For each study, the following data were extracted: (1) reference information (author, year); (2) sample characteristics (cell type, origin, number of donors); (3) PLA material and modifications (polymer type, specific modifications, material form); (4) comparison group(s); (5) main outcomes (reported quantitative and qualitative cellular responses); (6) authors’ conclusions; and (7) declared conflict of interest. A third author verified the accuracy and completeness of the extracted data, and any discrepancies were resolved through discussion to reach a consensus.

### 2.6. Data Items—Complementary

Characterization of materials—Standardized extraction of characterization parameters was performed for native and functionalized PLA/PLGA surfaces. The following features were recorded: topography (SEM, AFM; Ra/Rq metrics), surface chemistry (XPS/FTIR), wettability (contact angle), surface energy (Owens–Wendt method), crystallinity (XRD/DSC), zeta potential and mechanical properties, as well as coating stability when applicable.

Culture conditions—Extraction was standardized for cell type, seeding density, medium composition (serum/antibiotics), evaluation time points, incubation parameters (CO_2_/°C) and substrate preconditioning.

### 2.7. Risk of Bias Assessment

The Office of Health Assessment and Translation (OHAT) risk of bias (RoB) rating tool adaptation for in vitro studies was used to assess the RoB of the included studies. This tool was selected for its specific applicability to experimental in vitro studies, allowing for a structured evaluation of the credibility of the findings. The assessment focused on several key domains pertinent to the included studies, such as selection bias, confounding, characterization of the intervention (exposure), blinding of outcome assessment, and selective reporting of results [22].

The assessment was performed independently and in duplicate by two reviewers. To ensure consistent application of the tool’s criteria, the reviewers first conducted a calibration exercise on a subset of the included studies, supervised by a third author. For each domain within each study, a judgment of ‘low risk’, ‘unclear risk’, or ‘high risk’ of bias was assigned based on the signaling questions provided by the OHAT tool. Any disagreements between the two primary reviewers during the assessment process were resolved through discussion to reach a consensus. If an agreement could not be reached, the third author was consulted to make a final decision.

### 2.8. Outcome Measures

The primary outcomes were defined according to the cell type:

For osteoblasts and their progenitors: The assessment focused on cell viability, proliferation, cell adhesion and morphology, osteogenic differentiation (evaluated through markers such as alkaline phosphatase (ALP) and osteocalcin (OCN) expression), and extracellular matrix production and mineralization (e.g., evaluated by calcium deposition) [23].

For gingival fibroblasts: The outcomes of interest included cell viability, proliferation, cell adhesion, morphology, and migration, and extracellular matrix production (e.g., type I collagen) [24].

### 2.9. Data Synthesis

A narrative and qualitative synthesis of the included studies was conducted. Findings were thematically grouped according to cell type (osteoblasts/progenitors and gingival fibroblasts) and, within each group, were organized by type of outcome (e.g., adhesion, proliferation, differentiation). The results from each study were described systematically, highlighting the direction and magnitude of the observed effects and referencing the comparisons with relevant control groups.

## 3. Results

**Figure 1:**
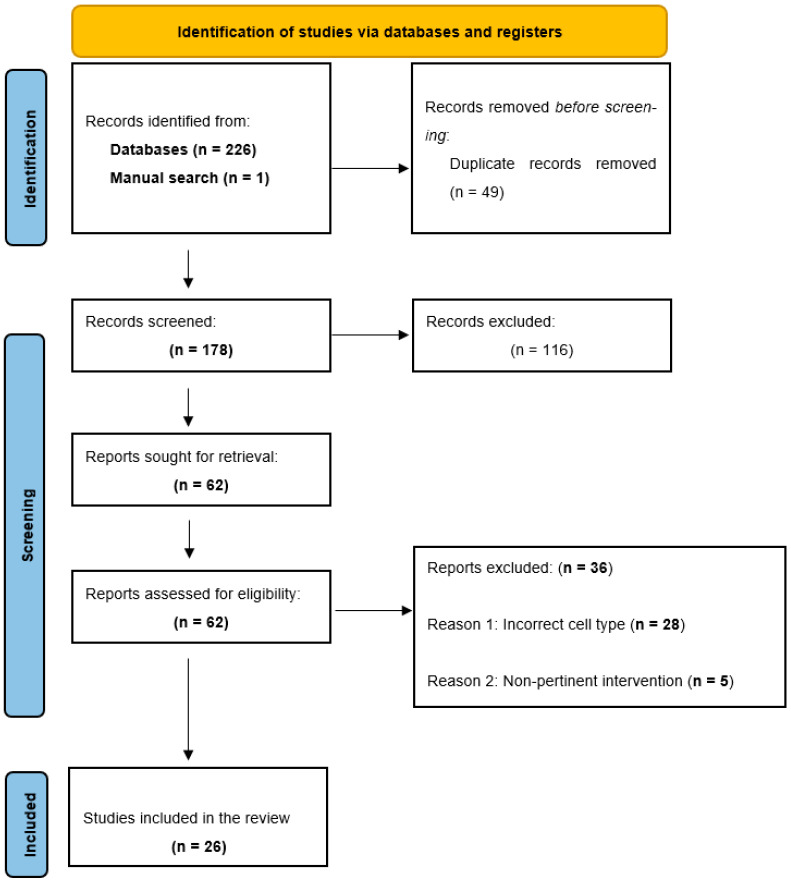
PRISMA 2020 flow diagram of the study selection process.

A total of 226 records were retrieved through the initial database search: Medline (n = 91), Scopus (n = 73), Web of Science (n = 60), and BVS (n = 2). After duplicate records removal (n = 49), 177 unique articles remained for screening. During title and abstract screening phase, 116 articles were excluded for being unrelated to the research question. Subsequently, the remaining 61 articles were selected for full-text review. In addition, 1 record was identified through manual searching; this record was also assessed in full and met the eligibility criteria. In total, 26 studies were included in the qualitative synthesis (Figure 1).

Flowchart depicting the studies selection for inclusion in the present systematic review.

### 3.1. Characteristics of the Included Studies

The 26 included studies were published between 1996 and 2023, reflecting a sustained and growing interest in the application of PLA-based materials for GBR. The studies originate from a diverse geographic range, with notable contributions from Asia (n = 12), Europe (n = 10), and North America (n = 4).

Among the included studies, 21 evaluated the response of primary human osteoblasts or their progenitor cells —including mesenchymal stem cells from bone marrow, adipose-derived stem cells, periodontal ligament, amnion, dental pulp, or skeletal sources—[25,26,27,28,29,30,31,32,33,34,35,36,37,38,39,40,41,42,43,44,45,46] (Table 1), while the remaining 5 studies focused on primary human gingival fibroblasts [31,47,48,49,50] (Table 2). The interventions were highly varied, ranging from unmodified polylactic acid-based materials, which were evaluated in 7 studies, to complex modifications. These included the creation of composites with bioceramics like hydroxyapatite (HA) or TiO_2_ [28,35,41], surface functionalization with inorganic coatings such as SiO_2_ or collagen [27,38]; alteration of microtopography through micro-grooves [31,49]; and loading with growth factors like BMP-2 or TGF-β1 [26,33,36]. The most common comparison groups were tissue culture polystyrene (TCPS) [30,32], unmodified polylactic acid-based materials, and other commercial membranes (e.g., ePTFE, collagen). Detailed information for each study is presented in Table 1 and Table 2.

### 3.2. Osteoblasts and Osteogenic Progenitors Response to Polylactic Acid-Based Materials

The response of osteoblastic lineage cells towards polylactic acid-based materials is strongly influenced by material formulation. A consistent trend shows that modifications to the polylactic acid-based materials matrix base, through the addition of bioceramics composites, the functionalization of its surface with coatings, and the incorporation of bioactive molecules like growth factors, are essential to enhance favorable cellular behavior [28,29,34,35]. Additionally, micro/nanotopographic patterning exemplified by PLA nanopillar arrays with controlled pillar diameters (≈100–300 nm; 450 nm pitch) can by itself steer osteogenic commitment of primary hADSCs without exogenous osteogenic supplements; notably, ≈200 nm pillars maximized ALP at day 14, upregulated RUNX2/OPN/OCN, enhanced mineralization in vitro, and improved ectopic osteogenesis versus planar PLA [46].

Characterization of materials

In the osteoblast/progenitor group, the most frequently used characterization technique was scanning electron microscopy (SEM) (n = 16; [27,28,29,30,31,32,33,34,35,36,38,39,42,43,44,45,46]). Less frequently, studies reported mechanical properties (n = 8; [29,33,37,38,39,41,43,45]), atomic force microscopy (AFM) roughness (Ra/Rq) (n = 4; [29,30,39,44]), contact angle (n = 3; [30,38,40]), X-ray photoelectron spectroscopy (XPS/ESCA) (n = 3; [29,30,44]), and X-ray diffraction (XRD) (n = 2; [29,35]). Fourier transform infrared spectroscopy (FTIR) was reported once (n = 1; [38]), and only a single study provided surface energy determined by the Owens–Wendt method (n = 1; [44]).

When key physicochemical metrics were reported (e.g., wettability/surface energy, AFM roughness Ra/Rq, and surface chemistry by XPS/ESCA), the trend was consistent: more hydrophilic surfaces with controlled sub-micrometric roughness were associated with more mature focal adhesions, higher ALP activity and RUNX2/OCN expression, and greater mineralization than native PLA/PLGA [29,30,33,35,38,40,44]. Strategies combining inorganic phases or bioactive coatings further reinforced these effects [27,29,30,33,35,38,44]. However, the low frequency of AFM/XPS reporting limits fine quantitative comparisons across studies.

Culture conditions

Culture conditions. Most studies incubated cells under standard conditions—37 °C and 5% CO_2_ (explicitly reported in n = 23; [25,26,27,28,29,30,31,32,33,34,35,36,37,38,39,40,41,42,43,44,45,46,49])—using either DMEM (n = 9; [28,31,33,34,36,38,39,41,44]) or α-MEM (n = 5; [32,33,35,38,46]) as basal media, supplemented with fetal bovine serum (FBS) (n = 14; [28,31,32,33,34,35,36,38,39,40,41,42,44,45,46]). Antibiotics/antimycotics (mostly penicillin–streptomycin) were added in n = 14 studies ([27,28,31,32,34,35,38,39,40,41,42,44,46,49]). Notably, Ref. [46] maintained osteogenic progenitors under growth-factor–free conditions.

Osteogenic induction using ascorbic acid, β-glycerophosphate, dexamethasone, or osteogenic medium was frequent (n = 14; [26,27,28,30,32,33,34,35,36,37,42,44,45,49]). While these approaches accelerate the RUNX2→OCN differentiation pathway and subsequent mineralization, they can create a ceiling effect that reduces detectable differences between surface conditions. By contrast, at least one study deliberately withheld osteogenic supplements to isolate material-driven effects; under growth-factor–free conditions, PLA nanopillar arrays still enhanced ALP, osteogenic gene expression, and mineralization [46].

Seeding density was reported in n = 13 studies ([25,26,27,30,33,34,35,36,37,38,39,41,44]) and acted as a confounder. High seeding densities favor paracrine signaling and early confluence, dampening sensitivity to micro/nanotopographic cues, whereas low densities amplify differences in adhesion and ALP activity.

Readouts were most often taken at day 7 (D7) (n = 10; [27,28,32,34,35,36,41,43,44,46]) and D14 (n = 6; [27,32,39,43,44,46]) for early events, and at D21 (n = 7; [27,32,39,43,44,46]) and D28 (n = 3; [34,43,44]) for mineralization. Thus, restricting observation to fewer than 14 days may fail to capture late effects of inorganic additives or PDA coatings; conversely, topography-driven responses can peak around D14 [46].

Substrate preconditioning was highly heterogeneous. Any form of preconditioning (n = 10; [27,30,32,33,36,37,38,39,42,44])—including protein coatings (collagen/fibronectin; n = 2; [25,30]), plasma treatment (n = 4; [30,39,42,44]), and PDA precoating (n = 6; [27,33,36,38,42,44])—tended to homogenize the initial protein-adsorption layer and was associated with faster cell attachment/spreading and higher osteogenic readouts, consistent with increased surface wettability and surface energy. Notably, one study intentionally avoided preconditioning and osteogenic supplements yet still observed enhanced ALP, osteogenic gene expression, and mineralization driven by nanoscale topography [46]. Nevertheless, under strong osteogenic induction, preconditioning may mask subtle differences attributable to the base surface properties.

Cell adhesion, spreading, and morphology:

Unmodified PLA clearly demonstrates limited suitability as a substrate for initial cell adhesion. For instance, Ellis and Chaudhuri reported that significantly fewer human bone-derived cells (HBDC) attached to pure PLA membranes at 6 h compared with tissue culture polystyrene (TCP) (*p* < 0.05), with the adhered cells exhibiting a predominantly rounded morphology suggesting a state of cellular stress and poor surface adhesion [30]. Similarly, Chen et al. noted restricted spreading and disorganized cytoskeletal organization of hMSCs on PLLA films, a specific PLA isomer [29].

In contrast, surface or compositional modifications to polylactic acid-based materials have been shown to significantly improve cell adhesion. In this line, studies by Terriza et al. showed that the functionalization of poly(lactic-co-glycolic) acid (PLGA) membranes—a copolymer derived from PLA—nanolayers of TiO_2_ or SiO_2_ via plasma-enhanced chemical vapor deposition significantly increased focal adhesion formation (*p* < 0.001), by inducing the reorganization of the actin cytoskeleton [37,38]. Likewise, Liu et al. reported a significant increase in osteoblast attachment on composites with 20 wt% nanophase titania compared to pure PLGA after 4 h of culture (*p* < 0.05) [28].

Cell proliferation

Evidence on cellular proliferation is mixed, suggesting that while the polylactic acid-based materials are cytocompatible, it does not consistently promote proliferation superior to standard controls. For instance, Ellis and Chaudhuri found significantly reduced proliferation of HBDCs on 50:50 and 75:25 PLGA membranes compared to TCP after 7 days (*p* < 0.05) [23]. In contrast, Hasan et al. observed no significant differences in DNA content between glass fiber-reinforced PLA composites and TCPs over a period of 14–21 days, indicating that certain formulations can provide adequate support for long-term cell proliferation [25].

Bioactive modifications appear to consistently improve the proliferative response to the PLA matrix. In this context, Fu et al. demonstrated that PLGA/nHA composite membranes yielded significantly higher CCK-8 absorbance values at 7 days than pure PLGA membranes (*p* < 0.05). This indicates a greater metabolic activity, which serves as a proxy for enhanced cell proliferation on the nano-hydroxyapatite functionalized surface [34].

Osteogenic differentiation and mineralization

This outcome is the most responsive to polylactic acid-based materials modifications, with the most compelling enhancements observed in bioactive formulations.

- ALP activity: polylactic acid-based materials demonstrate a capacity to maintain the osteoblastic phenotype. Marinucci et al. reported a 59% increase in ALP activity on Poly DL-lactide membranes compared to ePTFE controls [25]. However, bioactivation by incorporating composites or growth factors significantly amplifies this effect. Liu et al. observed a two- to three-fold increase in ALP activity on PLGA composites containing 30–40 wt% nanophase titania compared to pure PLGA at 21 days [28]. While Cho et al. reported an approximately five-fold increase of ALP levels in PLLA/Polydopamine/BMP-2 scaffolds compared to PLLA scaffolds alone at 14 days [26]. Additionally, a topography-only approach PLA nanopillar arrays (≈100–300 nm diameter; 450 nm pitch) under growth-factor free conditions produced the highest ALP on ≈200 nm pillars, peaking at day 14 [46].
- The expression of key osteogenic differentiation genes is markedly enhanced in modified scaffolds. Specifically, Wang et al. found that functionalizing PLGA/collagen nanofibers with the recombinant fusion peptide rFN/CDHs (Fibronectin/Cadherin) led to a significant upregulation of key osteogenic markers in hMSCs after 14 days: RUNX2 (~3-fold), ALP (~4.5-fold), and OCN (~5-fold) [45]. Similar patterns of osteogenic markers upregulations have been observed with BMP-2 or nHA incorporation into PLA/PLGA scaffolds in multiple studies using hMSCs or other osteoblastic progenitors (hAMSCs, hPDLSCs) [36,42,43,45].
- Matrix production and mineralization. Unmodified polylactic acid–based materials generally show minimal mineralization (Von Kossa or Alizarin Red). Ellis and Chaudhuri quantified <1 mineralized nodule/cm^2^ on PLGA membranes versus ~2.4 nodules/cm^2^ on TCPS [30]. In contrast, bioactive modifications promoted extensive calcium deposition [36,41]. Topography only PLA nanopillar arrays further increased mineralization and improved ectopic osteogenesis in vivo compared with planar PLA [46].

### 3.3. Gingival Fibroblasts Cells Response to PLA

The interaction of human gingival fibroblasts (HGFs) with PLA-based membranes is critical for soft tissue healing and maintenance of the barrier function during GBR.

Characterization of materials

In human gingival fibroblast (FGH) studies, characterization was more limited: SEM was reported (n = 2; [47,49]), along with single reports of contact angle (n = 1; [47]) and surface energy (n = 1; [49]). This limited dataset restricts the ability to correlate surface properties with adhesion and potential soft tissue sealing. Even so, studies reporting higher wettability consistently reported improved cell spreading and cytoskeletal organization than less hydrophilic or unmodified surfaces, supporting a more stable epithelial seal [47,49]. Integrin–FAK signaling on 3D-PLGA was also reported as a plausible substrate-mediated mechanotransduction pathway [50]. The near absence of AFM/XPS combined with the heterogeneity in seeding density and readout times may explain part of the interstudy variability.

Culture conditions

Reporting was sparse: DMEM and FBS were each specified (n = 1; [50]), antibiotics (n = 1; [47]), seeding density (n = 1; [47]), 37 °C (n = 2; [47,50]) and 5% CO_2_ (n = 1; [50]). Even so, studies that explicitly reported standard incubator conditions and serum supplementation described improved cell spreading and stress-fiber organization on more wettable or higher-energy surfaces, consistent with a more stable soft-tissue seal. However, the absence of explicit preconditioning, combined with heterogeneity in FBS concentration and seeding density and the lack of late (≥D21) readouts, limits robust correlation between culture handling and sealing behavior. Future studies should at minimum standardize serum concentration, seeding density, and evaluation windows (D7/D14 and ≥D21) to enable reproducible comparisons.

Adhesion, morphology, and migration

Early studies have consistently reported a poor fibroblast response to unmodified polylactic acid–based materials. Paynet observed no detectable migration (0.00 mm) of HGFs on PLA membranes, with cells adopting an abnormal “fried egg” morphology [47]. Similarly, Unsal et al., found significantly lower HGF attachment on PLA/PGA membranes (12.80 ± 0.47 cells/area) compared to collagen membranes (>21 cells/area) (*p* < 0.01) [48].

Surface modifications, however, have demonstrated the ability to modulate HGF behavior. Owen et al. showed that micro-grooved PLGA films induced an alignment of 80% of HGFs along the groove axis, despite reducing cell spreading area by ~35% [49]. More recently Wei et al. reported that HGFs cultured on a 3D PLGA scaffold under mechanical loading (25 g/cm^2^) reorganized their cytoskeleton and formed stress fibers, suggesting a functional and adaptive cell–material interaction in conditions that better simulate the vivo environment [18].

Proliferation and extracellular matrix production

HGF proliferation on smooth PLGA was comparable to standard controls but was significantly reduced (~40%) on micro-grooved films at day 7 [49]. In contrast, mechanical stimulation of HGFs on 3D PLGA scaffolds significantly upregulated type I collagen mRNA and protein expression by ~2-fold, indicating that scaffold architecture and biomechanical cues can enhance extracellular matrix deposition [50].

### 3.4. Risk of Bias of the Included Studies

The RoB assessment, conducted using the OHAT tool adapted for in vitro studies, revealed consistent methodological features across the included literature.

For the 21 studies evaluating osteoblasts and their progenitor cells, the overall risk of bias was judged to be low in domains related to material characterization, consistency of experimental conditions, and completeness of outcome reporting. Importantly, formal sample randomization was not considered critical in this context, since nearly all studies clearly stated that cells were treated under identical conditions, thereby minimizing the risk of systematic selection bias. Similarly, allocation concealment was rated as adequate whenever experimental groups were explicitly described, which was the case for the majority of the included works. The main limitation remained the lack of blinding in outcome assessment, which was rarely reported and thus represents a potential source of detection bias (Table 3).

The assessment was performed using the OHAT tool adapted for in vitro studies. Risk of bias was judged across key domains including exposure definition, allocation concealment, consistency of experimental conditions, blinding of outcome assessment, completeness of outcome data, selective reporting, and other potential sources of bias. Symbols represent the following: (++) low risk of bias; (+) probably low risk of bias; (-) high or unclear risk of bias.

The five studies that focused on human gingival fibroblasts displayed a comparable profile. As with the osteoblast studies, the experimental execution was generally robust, supported by clear descriptions of materials and conditions, while again showing deficiencies in blinding procedures (Table 4).

The same OHAT-based criteria were applied as in Table 3. Domains included allocation concealment, experimental consistency, blinding of outcome assessment, completeness of outcome data, selective reporting, and replication procedures. Symbols represent the following: (++) low risk of bias; (+) probably low risk of bias; (-) high or unclear risk of bias.

Finally, in the category of other sources of bias, a substantial proportion of the included studies reported conducting experiments in duplicate or triplicate. This practice enhances reproducibility and strengthens confidence in the reliability of their findings.

Taken together, this RoB assessment suggests that while the technical execution and reporting of in vitro studies on PLA-based materials are generally strong, the limited use of blinding and occasional lack of detail in replication procedures remain the primary methodological concerns.

## 4. Discussion

The main findings of this systematic review, derived from the analysis of 26 in vitro studies, conclude that polylactic acid and its copolymers are biocompatible, yet inherently bioinert. Their clinical potential for GBR is not fully realized in their unmodified form but is unlocked through targeted modifications that transform them into bioactive scaffolds capable of directing specific cellular responses. This marks a significant conceptual shift: from considering PLA as a passive barrier to recognizing it as a highly tunable platform for regeneration. The cumulated evidence suggests that targeted functionalization of PLA can be tailored to fulfill “dual bifunctionality” required for GBR: promoting osteogenesis on the side facing the defect, while guiding soft tissue integration on the external surface.

Across the 26 studies analyzed, several consistent patterns emerge that can inform evidence-based design strategies for optimizing osteogenic and soft-tissue responses. First, surface physicochemical properties appear to be a primary determinant of cell behavior: materials exhibiting increased wettability and surface energy, combined with controlled sub-micrometric roughness, consistently promote faster focal adhesion maturation and the upregulation of early osteogenic markers [29,30,33,35,38,40,44]. The incorporation of inorganic, osteoconductive phases such as calcium phosphates further enhances osteoblastic differentiation, as reflected by elevated ALP activity and calcium deposition compared to unmodified PLA/PLGA matrices [27,33,39]. Beyond compositional tuning, the mode of bioactive cue presentation also plays a decisive role. Covalent or polydopamine (PDA)-mediated immobilization of osteogenic molecules ensures stable and localized signaling, thereby amplifying differentiation and mineralization outcomes [28,44,45]. Interestingly, several reports demonstrate that even in the absence of exogenous supplements, nanoscale topographical features—particularly around 200 nm—can bias stem cell fate toward osteogenic lineages, emphasizing the instructive capacity of geometry itself [46]. Finally, at the soft-tissue interface, tailored micro-groove architectures and moderate surface energy were found to promote fibroblast alignment and robust adhesion while limiting excessive proliferation, contributing to the establishment of a stable and functional soft-tissue seal [47,49]. Collectively, these findings delineate a coherent set of design principles that transcend isolated experimental observations, providing a rational framework for the development of next-generation biomaterial surfaces supporting both hard- and soft-tissue integration.

Data from studies using human gingival fibroblasts indicate that the membrane’s outer face should be engineered to optimize submicrometric roughness and surface energy, thereby stabilizing the matrix-protein layer and promoting the formation of mature focal adhesions [50]. This fine-tuning of topography and surface chemistry regulates protein adsorption, cytoskeletal organization, and resistance to detachment under shear forces —factors that collectively determine the establishment of a stable soft-tissue seal [47,49,50]. However, the comparability across studies remains limited due to heterogeneity between seeding densities, evaluation times, and adhesion assessment methods, as well as the frequent omission of key features of the oral environment in most in vitro models [47,48,49,50].

The poor performance of unmodified PLA aligns with well-established principles of cell–biomaterial interaction. Indeed, the hydrophobic nature of PLA, assessed in different studies, hinders the adsorption of adhesion mediating proteins like vitronectin and fibronectin, which are essential for integrin-mediated cell attachment. This, at least partially, explains the poor spreading and rounded morphology of hMSCs and hBDCs observed by Chen et al., respectively [29]. Similarly, Payne et al. reported a complete inhibition of HGFs on PLA membranes, reinforcing the notion that PLA’s surface chemistry is suboptimal for initial cell attachment [47].

Long-term performance of PLA/PLGA-based materials is conditioned by hydrolytic and autocatalytic degradation, which generate an acidic microenvironment and local pH gradients. Progressive reductions in molecular weight and alterations in crystallinity can modify surface wettability and energy, directly influencing protein adsorption and the subsequent cellular responses. These physicochemical changes may attenuate or reverse the initial benefits of coatings and inorganic fillers observed during short culture periods. Accordingly, future studies should incorporate extended follow-up periods, monitor pH and lactate accumulation, while document coating stability together with the retention of mechanical and surface properties. This approach will help identify modification strategies that maintain their effects under realistic conditions of polymer degradation [4,7,8].

The success of PLA modification strategies can be interpreted through their ability to overcome these early shortcomings. By modifying the surface chemistry and topography, Terriza showed that coating PLA with a thin nanolayer of TiO_2_ or SiO_2_ is sufficient to significantly enhance osteoblast adhesion and cytoskeletal organization (*p* < 0.001) [30,31]. Similarly, Owen et al., demonstrated that micro-topographic cues alone can direct HGF alignment [41], while Graziano et al., found such cues may even induce osteogenic differentiation in DPSCs [31]. These suggests that the initial cell “sensing” of the surface chemistry is a critical modulating event. On the other side, the incorporation of bioactive cues showed the most dramatic osteogenic responses when bioceramics like HA or growth factors like BMP-2 were incorporated [41].

Topography-only evidence. Beyond chemical or biochemical functionalization, nanoscale geometry alone can be instructive. In the topography-only study [46], PLA nanopillar arrays (≈100–300 nm diameter; 450 nm pitch) induced osteogenic commitment of primary hADSCs under growth-factor–free conditions. Pillars of ≈200 nm maximized ALP around day 14, up-regulated RUNX2/OPN/OCN with increased Alizarin Red mineralization by day 21 and enhanced ectopic osteogenesis versus planar PLA. These findings reinforce that surface nanotopography can drive fate decisions independently of biochemical cues and dovetail with the dual-biofunctionality design principle for GBR membranes. Nevertheless, this evidence comes from a single primary cell source and short timeframes with limited blinding, warranting validation across donors and under conditions that simulate hydrolytic degradation.

Liu et al. reported a 2- to 3-fold increase in ALP activity with titania composites [28], while Cho et al. observed a 5-fold increase when BMP-2 was immobilized on PLA scaffolds [33]. These findings are consistent with the known osteoconductive properties of calcium phosphates, by providing nucleation sites for mineralization, and the potent osteogenic potential of BMP-2, thus highlighting a shift from a merely osteoconductive to an actively osteoinductive material.

Despite the overall consistency, some variability in results warrants further discussion. For instance, while Ellis and Chaudhuri reported reduced proliferation of human bone-derived cells on PLGA membranes compared to controls [30], Hasan et al. observed comparable long-term proliferation on PLA composites [32]. This could be explained due to differences in degradation kinetics, where faster degradation of the PLGA membranes might have led to a more acidic microenvironment that temporarily inhibited proliferation, an effect less pronounced in more stable PLA composites. Moreover, heterogeneity between primary cells sources may also account for inherent biological variability affecting the magnitude of the observed effects across studies. Otherwise, an important unresolved question is the long-term impact of acidic degradation by products on mature, mineralized matrix integrity, an issue not addressed by the included short-term studies.

The findings of this review carry practical implications for the design of next-generation GBR membranes. The cumulated evidence suggests that passive barriers should no longer be the gold standard, and that GBR membrane must be engineered instead with a dual-sided functionality. For instance, an internal surface functionalized with HA or a low dose of BMP-2, to actively recruit and promote the differentiation osteoprogenitor cells [36,41]. And complementarily, an external surface with a tailored micro-topography to align gingival fibroblasts and limit over-proliferation [49], potentially enhancing flap stability and minimizing fibrous encapsulation. As Chen et al. notes, it is essential to distinguish between statistically significant and clinically meaningful differences. While minor increases in ALP activity may not directly impact clinical performance, the multi-fold increases observed with functionalized PLA modifications are more likely to translate into clinically relevant improvements in bone regeneration [29].

While this review synthesized a valuable body of in vitro evidence, several limitations must be acknowledged. The most significant limitation is the high methodological heterogeneity across studies, which prevented the performance of a pooled quantitative analysis. There is a clear lack of standardization in cell culture protocols, the specific formulation and characterization of PLA materials, and the time points chosen for evaluation. Secondly, the risk of bias assessment revealed that many studies failed to report on key methodological aspects, particularly the blinding of outcome assessment, introducing potential for detection bias. Finally, and most critically, these are all in vitro studies. Despite providing invaluable mechanistic insights, these cannot fully replicate the complexity of the in vivo environment, including the immune response, angiogenesis, and mechanical loading.

Therefore, to elaborate on the state of the evidence and address the current gap, future research should prioritize: the development of guidelines for the in vitro testing of membranes for GBR, including a minimum set of material characterization and cellular outcomes to be reported, which would improve their comparability and reliability [29]. The RoB assessment indicates that most in vitro studies on PLA-based materials demonstrate strong technical execution and consistent reporting, particularly in material characterization and standardized experimental conditions. However, the limited reporting of blinding procedures and occasional lack of detail regarding replication remain important considerations, underscoring the need for greater methodological transparency in future research. Moreover, further studies should move towards more complex cellular models, such as co-cultures osteoblast-fibroblast on opposite sides of a test membrane, or macrophage-inclusive of to assess the material’s inflammatory response profile [51]. In this context, the most promising modifications, such as those combining bioceramic composites with growth factors or specific topographies, should undergo rigorous validation in preclinical GBR animal models to evaluate the cellular regenerative performance under physiologic conditions.

This review was deliberately restricted to primary human cells to maximize the physiological relevance of in vitro outcomes in the context of GBR and to reduce methodological artifacts. Immortalized cell lines were excluded because they may exhibit genomic alterations and non-physiological proliferation rates that distort adhesion, ALP, osteogenic marker expression (RUNX2, OCN), and mineralization, compromising the validity of comparisons among PLA/PLGA-based surfaces [14,52]. Accordingly, the methodological scope was defined to obtain mechanistic inferences with higher biological validity in humans and comparable outcomes on native and functionalized PLA/PLGA surfaces, acknowledging that clinical translation will require subsequent preclinical validation in appropriate models.

The next generation of polylactic acid–based biomaterials for GBR should adopt a more explicitly translational approach, emphasizing human-relevant in vitro standards. This includes harmonizing donor reporting and using canonical time points that mirror the temporal dynamics of GBR in clinical settings. Technically, adopting a core outcome set—encompassing cell adhesion and focal adhesion maturation, ALP activity, RUNX2 and OCN expression, and matrix mineralization—would enable more robust comparisons of effect sizes across studies. Technically, adopting a core outcome set —including cell adhesion and focal adhesion maturation, ALP activity, RUNX2 and OCN expression, and matrix mineralization—would enable more robust comparisons of osteogenic effect sizes across studies. Equally important is the explicit evaluation of two-sided membrane designs that decouple bone-facing osteoinductivity from soft-tissue sealing, for instance through asymmetric topographies or surface chemistries. Another key priority is the rigorous quantification of coating and functionalization stability under physiologic conditions, linking retention over time to degradation kinetics, pH shifts, and lactic acid release, and correlating these parameters with cellular responses. Finally, future studies should incorporate immunoregulatory and tissue cross-talk models, such as macrophage–osteoblast and fibroblast–epithelial co-cultures, to better capture early immune polarization, soft-tissue sealing dynamics, and the influence of surface energy and topography on these intercellular processes.

## 5. Conclusions

This systematic review of in vitro studies using primary human cells shows that polylactic acid-based materials are cytocompatible but relatively bioinert when compared with functionalized surfaces or matrices. Incorporating inorganic phases, optimizing surface topography and energy, and immobilizing or releasing signaling factors into PLA consistently enhance osteoblasts and progenitors’ responses—including cell adhesion, alkaline phosphatase activity, osteogenic differentiation (RUNX2 and OCN expression), and matrix mineralization—compared with native PLA. For human gingival fibroblasts, the available evidence supports its cytocompatibility and potential for soft-tissue sealing, although the body of comparable data is smaller and more heterogeneous.

Persistent knowledge gaps concern the long-term stability of surface modifications and the biological effects of polymer degradation products beyond short-term culture experiments. Taken together, these data support the development of barrier membranes with dual biofunctionality as a promising strategy for guided bone regeneration. Future research should consider standardized outcome measures, followed by its validation applying adequate in vivo GBR models in maxillary bones before clinical translation.

### Take-Home Messages

- PLA and PLGA are biocompatible but exhibit limited osteogenic potential without functional modification.
- Inorganic modifications and controlled surface properties improve adhesion, alkaline phosphatase activity, osteogenic differentiation and mineralization.
- Human gingival fibroblasts show compatibility with polylactic acid-based materials, but the evidence base is small and heterogeneous.
- Long-term data on polylactic acid-based materials modifications stability and the effects of acidifying degradation of the polymer are lacking.

## Figures and Tables

**Table 1:** Summary of included studies evaluating the response of osteoblasts and osteogenic progenitor cells to PLA-based materials.

Reference	Sample	Polylactic Acid-Based Material and Modifications	Comparison	Main Outcomes	Authors’ Conclusions	Conflict of Interest
Marinucci, L. et al. (2001) [25]	Human osteoblasts *Origin*: Jaw fragments from third molar surgery *Donors*: 4	*PLA Type*: Poly DL-lactide *Form*: Membrane	ePTFE (non-resorbable), collagen and hyaluronic acid membranes	**Proliferation (^3^H-thymidine):** Poly DL-lactide stimulated DNA synthesis 3.1-fold more than ePTFE **Differentiation (ALP):** Increased ALP activity by 59% vs. ePTFE **ECM Prod (^3^H-proline):** Increased collagen synthesis (1.9-fold vs. ePTFE)	Bioabsorbable membranes, including PLA, appear to promote bone regeneration through their activity on osteoblasts	Not reported.
Lilli, C. et al. (2002) [26]	Human osteoblasts Origin: Jaw fragments from orthodontic surgery N donors: 4	PLA Type: Poly DL-lactide Modification: Enriched with TGF-β1	Untreated PLA membrane; other commercial membranes (PTFE, Paroguide, HYAFF)	**Proliferation (^3^H-thymidine):** No significant effect of TGF-β1 on DNA synthesis (*p* > 0.05) **Differentiation (ALP, OCN):** TGF-β1 enriched PLA significantly increased ALP activity (~1.3-fold) and osteocalcin secretion (~2-fold) (*p* < 0.05)	Membranes enriched with TGF-β1 stimulated osteoblastic parameters more than untreated membranes	Not reported.
Yang, Y. et al. (2002) [37]	human osteoblasts Origin: Trabecular bone from tibial fractures	PLA Type: PLLA Modification: Scaffold loaded with calcium channel agonist (BAY K8644) + collagen coated	PLLA/Collagen scaffold without agonist; static vs. mechanical loading conditions	**Viability (LDH):** No significant differences observed **Differentiation (ALP/protein):** Significantly enhanced (>2-fold) with BAY K8644 under load (*p* < 0.05) **ECM Prod. (Collagen I):** Enhanced with BAY K8644 under load (*p* > 0.05 at 24 h)	Manipulating calcium channels via a mechano-active scaffold is an effective technique to amplify matrix production in response to mechanical stimulation	Not reported.
Liu, H. et al. (2006) [39]	human osteoblasts Origin: Femoral heads from hip replacement surgery	PLA Type: PLGA. Modification: Composite with 0–40 wt% nanophase titania	Control Group(s): Pure PLGA; PLGA with conventional titania	**Adhesion and Proliferation:** Adhesion, determined by counting the number of attached cells after 4 h, and proliferation, measured by quantifying total cellular DNA content at various time points, were significantly greater on composites with >20% nanophase titania (*p* < 0.05) **Differentiation (ALP, Mineralization):** Differentiation was assessed by measuring ALP activity through a colorimetric assay and quantifying calcium deposition. Results showed significantly higher ALP activity (2–3 fold) and calcium deposition on composites with 30–40% titania (*p* < 0.01)	Increasing nanoscale surface roughness by adding nanophase titania to PLGA selectively enhances long-term osteoblast adhesion, proliferation, and function	Not reported.
Chen, Y. et al. (2008) [40]	Human mesenchymal stem cells (hMSC) Origin: Bone marrow from healthy donors	PLA Type: PLLA Form: Films	Control Group(s): Apatite and apatite/collagen coated surfaces	**Adhesion/Morphology (SEM, Immunofluorescence):** hMSCs showed poor spreading, a rounded morphology, and less organized focal adhesions and stress fibers on PLLA surfaces compared to apatite-containing surfaces. *p* values not reported	The PLLA surface was not favorable for the adhesion and spreading of hMSCs compared to apatite and apatite/collagen surfaces	Not reported.
Ellis, M.J. & Chaudhuri, J.B. (2008) [41]	Human bone derived cells (HBDC) Origin: Femoral head from a 29-year-old female donor	PLA Type: PLGA with L:G ratios of 50:50, 75:25, and 100:0 (pure PLA)	Control Group(s): Tissue culture polystyrene (TCPS)	**Adhesion (DNA, 6 h):** 100:0 PLA had significantly fewer cells than TCPS (*p* < 0.05) Proliferation (DNA, 7 d): 50:50 and 75:25 PLGA had significantly fewer cells than TCPS (*p* < 0.05), but 100:0 PLA was comparable (*p* > 0.05). **Differentiation (Mineralization):** All membranes showed significantly less mineralization (<1 nodule/cm^2^) than TCPS (~2.4 nodules/cm^2^) (*p* < 0.05)	PLGA membranes of various ratios support HBDC culture; the optimal ratio can be selected based on other factors like degradation rate	Not reported.
Graziano, A. et al. (2008) [42]	Human dental pulp stem cells (DPSC) Origin: Third molars	PLA Type: PLGA (85:15) Modification: Micro-grooved surface topography	Control Group(s): PLGA scaffold with a flat surface	**Differentiation (Immunofluorescence):** DPSCs differentiated into osteoblasts, formed a 3D matrix, and expressed bone proteins (osteopontin, osteonectin, BSP) only on the micro-grooved scaffolds, not on the flat ones. *p* values not reported	The micro-grooved topography on PLGA scaffolds was sufficient to induce the osteoblastic differentiation of human dental pulp stem cells	Not reported.
Hasan, M.S. et al. (2013) [43]	Human osteoblasts Origin: ECACC (commercial)	PLA Type: PLA matrix Modification: Reinforced with phosphate glass fibers (PGF) treated with various coupling agents	Control Group(s): Composite with untreated fibers; TCPS	**Proliferation (DNA):** No significant difference in cell growth between all composite groups and controls over 21 days (*p* > 0.05). **Differentiation (ALP, OCN, Collagen):** Normal differentiation trends comparable to controls (*p* > 0.05), with no adverse effects from the coupling agents	All composites, regardless of the fiber treatment, demonstrated cytocompatibility comparable to controls, supporting their use in implantable devices	The authors declare no conflicts of interest.
Cho, H.J. et al. (2014) [44]	human mesenchymal stem cells (hMSCs) Origin: Lonza (commercial)	PLA Type: PLLA Modification: Nanofibers coated with polydopamine (PDA) to immobilize BMP-2	Control Group(s): Unmodified PLLA nanofibers and nanofibers coated only with PDA	**Differentiation (ALP, Alizarin Red):** Immobilization of BMP-2 on PLLA nanofibers significantly promoted osteogenic differentiation ALP activity was approximately 2.1-fold higher on the BMP-2 immobilized group compared to the unmodified PLLA group (*p* < 0.05), and matrix mineralization, confirmed by Alizarin Red S staining, was also significantly increased (*p* < 0.05)	Polydopamine-mediated immobilization of BMP-2 on PLLA nanofibers is an effective method for enhancing osteogenic differentiation	Not reported.
Wang, J. et al. (2014) [45]	human bone marrow mesenchymal stem cells (hBMSCs) Origin: ScienCell (commercial)	PLA Type: PLGA Modification: Core-shell nanofibers of PLGA/collagen loaded with rFN/CDHs peptides	Control Group(s): PLGA/collagen nanofibers without peptides	**Proliferation (CCK-8):** Loading with peptides significantly enhanced hBMSC proliferation in a dose-dependent manner (*p* < 0.05 or *p* < 0.01) **Differentiation (RT-qPCR):** Cells cultured on scaffolds with rFN/CDHs expressed significantly higher levels of osteogenic genes compared to scaffolds without rFN/CDHs. At 14 days, the 50 µg/mL concentration group showed a ~4.5-fold increase in ALP, a ~3-fold increase in RUNX2, and a ~5-fold increase in OCN expression (*p* < 0.0001 for all)	PLGA/collagen nanofiber scaffolds loaded with rFN/CDHs are a promising substrate for bone tissue engineering	The authors report no conflict of interest.
Tayton, E. et al. (2014) [27]	human skeletal stem cells (hSSCs) Origin: Bone marrow aspirates	PLA Type: PLA and PLGA Modification: Composites with HA	Control Group(s): Comparison between PLA vs. PLGA and pure polymer vs. composite with HA	**Viability (Live/Dead):** High cell viability in all scaffolds **Differentiation (ALP/DNA):** PLGA + HA scaffolds showed the highest osteoblastic activity at day 14. ECM Prod.: Strong collagen-1 staining in all groups. *p* values not reported	All four types of polymeric scaffolds are biocompatible and osteoconductive, with PLGA-HA composites showing the highest osteoblastic activity in vitro	The authors declare no conflict of interest.
Beazley, K.E. et al. (2014) [28]	human bone marrow-derived mesenchymal stem cells (hBMSCs) Origin: Lonza (commercial)	PLA Type: PLLA Modification: PLLA/Collagen scaffold with covalently cross-linked BMP-2	Control Group(s): Scaffold with physically adsorbed BMP-2	**Differentiation (qPCR, Alizarin Red):** Cross-linking of BMP-2 promoted superior osteogenic differentiation (significant increase in osteogenic genes, *p* < 0.05) and a more calcified matrix compared to simple adsorption.	Covalent cross-linking of BMP-2 to collagen-PLLA scaffolds is an effective strategy to promote osteogenic differentiation of hBMSCs	The authors declare no conflict of interest.
Terriza, A. et al. (2014) [29]	Normal Human Osteoblasts (HOB^®^). Origin: Promocell (commercial)	PLA Type: PLGA Modification: Coated with a TiO2 nanolayer (10–100 nm) by PECVD	Control Group(s): Uncoated PLGA	**Adhesion/Morphology (Phase contrast, Immunofluorescence):** The TiO_2_ coating significantly improved cell spreading, elongation, and the development of focal adhesions (*p* < 0.001), in a layer thickness-dependent manner	The deposition of TiO_2_ by PECVD is a valuable tool to increase the bioactivity of PLGA membranes, enhancing the osteoblastic response	The authors declare no conflict of interest.
Terriza, A. et al. (2014) [30]	Primary Human Osteoblasts (HOB). Origin: Promocell (commercial)	PLA Type: PLGA Modification: Coated with a thin film (15 nm) of SiO_2_ by PECVD	Control Group(s): Uncoated PLGA	**Adhesion/Morphology (Phase contrast, Immunofluorescence):** Improved cell spreading, elongation, and intercellular contacts on SiO_2_-coated membranes. Significant increase in focal adhesions (*p* < 0.001)	The SiO_2_ coating by PECVD is a biocompatible method that elicits a significant osteoblastic response on PLGA membranes	The authors declare that there is no conflict of interests.
Raghavendran, H.R.B. et al. (2016) [31]	Primary human mesenchymal stromal cells (hMSCs). Origin: Bone marrow of healthy donors	PLA Type: PLLA Modification: PLLA/Col/HA, PLLA/HA, PLLA/Col scaffolds, treated with PDGF	Control Group(s): Scaffolds not treated with PDGF	**Differentiation (Alizarin Red, qPCR):** PDGF treatment of the PLLA-based scaffolds produced rapid and enhanced osteogenic differentiation, with greater mineralization and OCN expression (*p* < 0.05)	PDGF interacts synergistically with the surface of PLLA-based scaffolds to produce rapid osteogenic differentiation of hMSCs	The authors declare no competing financial interest.
Guduric, V. et al. (2017) [32]	Primary human bone marrow stromal cells (HBMSCs). Origin: Bone marrow aspirates from healthy donors	PLA Type: PLA. Form: Porous membranes fabricated by 3D printing	Control Group(s): Static culture vs. dynamic perfusion	**Viability, Proliferation, Differentiation (ALP, Alizarin Red, qPCR):** The 3D printed PLA membranes supported HBMSC viability, proliferation, and osteoblastic differentiation (significant gene upregulation vs 2D culture), especially under dynamic perfusion conditions. *p* values not reported	3D printed PLA membranes are a suitable substrate for bone tissue engineering, and their performance can be enhanced by culture in perfusion bioreactors	The authors declare that they have no conflict of interest.
Fu, L. et al. (2017) [33]	Primary human bone marrow mesenchymal stem cells (hBMSCs) Origin: Cyagen Biosciences (commercial)	PLA Type: PLGA Modification: Bilayer membrane of PLGA/nano-Hydroxyapatite (nHA)	Control Group(s): Pure PLGA membrane; culture wells	**Adhesion, Proliferation, and Differentiation:** The bilayer PLGA/nHA membrane significantly improved (*p* < 0.05) adhesion, proliferation, and osteogenic differentiation of hBMSCs compared to pure PLGA	The bilayer PLGA/nHA membrane is a promising candidate for GBR due to its excellent bioactivity and promotion of osteogenesis	The authors declare no conflict of interest.
Wu, S. et al. (2018) [34]	Primary human amnion mesenchymal stem cells (hAMSCs) Origin: Human placentas	PLA Type: PLA Modification: nHAC/PLA scaffold loaded with BMP-2	Control Group(s): nHAC/PLA scaffold without BMP-2	**Proliferation and Differentiation:** Loading BMP-2 onto the nHAC/PLA scaffold significantly enhanced the proliferation and osteogenic differentiation (OCN and Runx2 expression) of hAMSCs (*p* < 0.05)	nHAC/PLA scaffolds loaded with BMP-2 are a promising biomaterial for bone tissue engineering	The authors declare no conflict of interest.
Kang, I.-G. et al. (2020) [35]	Primary human bone marrow-derived mesenchymal stem cells (hBMSCs) Origin: Lonza (commercial)	PLA Type: PLLA Modification: PLLA membrane with embedded HA patterns loaded with rhBMP-2	Control Group(s): Unmodified PLLA membrane; PLLA with HA patterns but no BMP-2	**Differentiation:** The HA patterns loaded with BMP-2 on the PLLA membrane effectively promoted osteogenic differentiation of hMSCs, showing higher ALP activity and gene expression compared to controls. *p* values not reported	Creating HA patterns loaded with growth factors on PLLA membranes is an effective strategy to enhance osteogenic bioactivity	The authors declare no conflict of interest.
Chen, Y. et al. (2020) [36]	Primary human mesenchymal stem cells (hMSCs) Origin: ATCC (commercial)	PLA Type: PLGA Modification: Hybrid meshes of PLGA-collagen-ECM	Control Group(s): Pure PLGA; PLGA-collagen	**Differentiation:** The hybrid meshes incorporating decellularized ECM significantly enhanced osteogenic differentiation of hMSCs, as demonstrated by statistically higher alkaline phosphatase (ALP) activity, calcium deposition (Alizarin Red S), and expression of osteopontin (OPN) and osteocalcin (OCN) compared to the PLGA and PLGA-collagen scaffolds (*p* < 0.01 for all comparisons)	Hybrid PLGA-collagen-ECM meshes that mimic the composition of the developing bone matrix are a promising scaffold for bone tissue engineering	The authors declare that they have no competing interests.
Zhang, Y. et al. (2023) [38]	Primary human periodontal ligament stem cells (hPDLSCs) Origin: Extracted healthy premolars	PLA Type: PLA Modification: Janus membrane with a metal-phenolic network (MPN) on one side	Control Group(s): Unmodified PLA membrane	**Adhesion/Morphology (SEM):** Better adhesion and spreading on the MPN-modified side. Differentiation (ALP, Alizarin Red, RT-qPCR): The modified side significantly promoted osteogenic differentiation (higher expression of RUNX2, ALP, OPN, OCN) (*p* < 0.01)	The Janus PLA membrane with a metal-phenolic interface promoted osteogenic differentiation of hPDLSCs in vitro	The authors declare no competing interests.
Zhang, S. et al. (2018) [46]	Primary human adipose-derived stem cells (hADSCs); source: Qilu Hospital, Shandong University.	PLA nanopillar arrays fabricated by AAO-template nanoimprint; pillar diameters 100/200/300 nm, same center-to-center distance 450 nm, ~100 nm height; growth-factor–free culture (no osteogenic supplements).	PLA planar film and tissue-culture plates (TCP/TCPS) as controls.	**Adhesion/morphology:** Distinct cytoskeletal organization and morphology across diameters; PLA-200 yielded polygonal, osteoblast-like morphology and higher vinculin signal vs. planar PLA/PLA-100. Differentiation (ALP): Peak ALP at day 14 on all samples, highest on PLA-200; ALP decreased by day 21 (early marker). **Genes/Mineralization:** qPCR of RUNX2/OPN/OCN and Alizarin Red S mineralization assessed at 7/14/21 days (greater osteogenic readouts on nanopillars; strongest overall on PLA-200).	Nanopillar diameter is a critical design variable; ~200 nm pillars can drive osteogenic differentiation of hADSCs without growth factors and enhance ectopic bone formation	The authors declare no competing interests.

For each article, the table details the reference, cell sample characteristics, the type of PLA material and its modifications, the comparison groups, the main outcomes, and the authors’ conclusions. To facilitate readability, the following abbreviations are defined: **ALP** (Alkaline Phosphatase), **BMP-2** (Bone Morphogenetic Protein-2), **ECM** (Extracellular Matrix), **ePTFE** (expanded Polytetrafluoroethylene), **HA** (Hydroxyapatite), **hAMSCs** (human Amnion Mesenchymal Stem Cells), **hBMSCs** (human Bone Marrow Mesenchymal Stem Cells), **hMSCs** (human Mesenchymal Stem Cells), **hPDLSCs** (human Periodontal Ligament Stem Cells), **hSSCs** (human Skeletal Stem Cells), **nHA** (nano-Hydroxyapatite), **OCN** (Osteocalcin), **PECVD** (Plasma-Enhanced Chemical Vapor Deposition), **PDGF** (Platelet-Derived Growth Factor), **PLLA** (Poly-L-lactic Acid), **PLGA** (Poly(lactic-co-glycolic) acid), **RUNX2** (Runt-related transcription factor 2), **SiO_2_** (Silicon Dioxide), **TCPS** (Tissue Culture Polystyrene), **TGF-β1** (Transforming Growth Factor-beta 1), and **TiO_2_** (Titanium Dioxide).

**Table 2:** Summary of Included Studies Evaluating the Response of Human Gingival Fibroblasts to PLA-Based Materials.

Reference	Sample	Polylactic Acid-Based Materials and Modifications	Comparison	Main Outcomes	Authors’ Conclusions	Conflict of Interest
Payne, J.M. et al. (1996) [47]	Primary human gingival fibroblasts (HGF). Origin: Retromolar tissue explants	PLA Type: Polylactic acid (Atrisorb^®^ membrane)	Control Group(s): Polystyrene; ePTFE and calcium sulfate membranes	**Adhesion/Morphology and Migration:** Cells exhibited an abnormal “fried egg” morphology on the PLA surface. The corrected migration distance on PLA was 0.00 mm, which was significantly less than that on polystyrene (1.72 mm) (*p* < 0.001)	PLA membranes exhibited a morphology not conducive to migration or cell health in this in vitro model	Not reported.
Ünsal, B. et al. (1999) [48]	Primary human gingival fibroblasts (HGF). Origin: Retromolar tissue explants	PLA Type: PLA/PGA copolymer (Resolut^®^ membrane)	Control Group(s): Four types of commercial collagen membranes	**Adhesion/Morphology:** Initial cell attachment was significantly lower (*p* < 0.01) on the PLA/PGA membrane (12.80 ± 0.47 cells/area) vs. all collagen membranes (>21 cells/area)	Collagen-based membranes offer greater potential for initial fibroblast attachment than PLA-based membranes	Not reported.
Owen, G.R. et al. (2005) [49]	Primary human gingival fibroblasts (HGF). Origin: Healthy gingival tissue explants	PLA Type: PLGA (85:15) Modification: Micro-grooved surface topography	Control Group(s): PLGA films with a smooth surface	**Adhesion/Morphology:** Micro-grooves induced strong alignment (>80% of cells) but reduced cell spreading area. Proliferation: Significantly reduced (~40% less at day 7) on grooved surfaces (*p* < 0.05)	Surface topography can effectively control fibroblast behavior, which could be beneficial for GTR applications	Not reported.
Graziano, A. et al. (2008) [42]	Primary human gingival fibroblasts. Origin: Gingival biopsies.	PLA Type: PLGA (85:15) Modification: Micro-grooved surface topography	Control Group(s): PLGA scaffold with a flat surface	**Adhesion/Morphology and Differentiation:** HGFs were used as a negative control. They adhered to the PLGA scaffolds but showed no signs of osteogenic differentiation, regardless of the topography. *p* values not reported	Gingival fibroblasts do not differentiate into an osteoblastic phenotype on PLGA scaffolds, even with topographical cues.	Not reported.
Wei, L. et al. (2020) [50]	Primary human gingival fibroblasts (HGFs). Origin: Healthy premolars from patients aged 10–14 N donors: 15	PLA Type: PLGA Modification: Application of compressive mechanical force (25 g/cm^2^)	Control Group(s): Cells on scaffold without mechanical force	**ECM Prod. (Collagen I):** Force significantly increased collagen expression (~2-fold in mRNA at 24 h peak) (*p* < 0.05). Gene/Protein Expression: Force significantly increased integrin α_5_β_1_ and FAK expression (*p* < 0.05)	The integrin α_5_β_1_/FAK signaling pathway and the cytoskeleton are involved in HGF mechanotransduction	The authors declare no conflicts of interest.

For each study, the table presents the reference, cell sample characteristics, the type of PLA material evaluated and its modifications, the comparison groups, the main outcomes obtained, and the conclusions reported by the authors. To facilitate readability, the following abbreviations are defined: **ECM** (Extracellular Matrix), **ePTFE** (expanded Polytetrafluoroethylene), **FAK** (Focal Adhesion Kinase), **HGFs** (Human Gingival Fibroblasts), **PGA** (Polyglycolic Acid), and **PLGA** (Poly(lactic-co-glycolic) acid).

**Table 3:** Risk of bias assessment for the included studies that analyzed the response of osteoblasts and their progenitor cells.

	Risk of Bias								
Study	Randomization	Allocation Concealment	Experimental Conditions	Blinding During Study	Incomplete Data	Exposure Characterization	Outcome Assessment	Selective Reporting	Other Sources of Bias
Marinucci L. et al. [25]	+	++	++	-	++	++	-	++	++
Lilli C. et al. [26]	+	++	++	-	++	++	-	++	++
Ellis MJ. et al. [41]	+	-	++	-	++	++	-	++	++
Terriza A. et al. [29]	+	++	++	-	++	++	-	++	++
Terriza A. et al. [30]	+	-	++	-	++	++	-	++	++
Fu L. et al. [33]	+	++	++	-	++	++	++	++	++
Hasan MS. et al. [43]	+	-	++	-	++	++	-	++	++
Yang Y. et al. (2002) [37]	+	-	++	-	++	+	-	++	-
Liu H. et al. (2006) [39]	+	-	++	-	++	++	+	++	++
Chen Y. et al. (2008) [40]	+	-	++	-	++	+	-	++	-
Graziano A. et al. (2008) [42]	+	+	++	-	++	++	-	++	++
Cho HJ. et al. (2014) [44]	+	+	++	-	+	+	-	+	+
Wang J. et al. (2014) [45]	+	-	++	-	++	++	-	++	++
Tayton E. et al. (2014) [27]	+	+	++	-	++	++	-	++	++
Beazley KE. et al. (2014) [28]	+	-	++	-	++	+	-	++	-
Raghavendran et al. (2016) [31]	+	-	++	-	++	+	+	++	++
Guduric V. et al. (2017) [32]	+	+	++	-	++	++	-	++	++
Wu s. et al. (2018) [34]	+	+	++	-	++	++	-	++	++
Kang IG. et al. (2020) [35]	+	+	++	-	++	++	-	++	++
Zhang Y. et al. (2023) [38]	+	++	++	+	++	++	+	++	++
Zhang S. et al. (2018) [46]	+	-	+	-	++	++	+	++	+

**Table 4:** Risk of bias assessment for the included studies that analyzed the response of human gingival fibroblasts.

	Risk of Bias								
Study	Randomization	Allocation Concealment	Experimental Conditions	Blinding During Study	Incomplete Data	Exposure Characterization	Outcome Assessment	Selective Reporting	Other Sources of Bias
Payne JM. et al. [47]	+	++	++	-	++	++	-	++	-
Ünsal et al. [48]	+	++	++	-	++	++	++	++	-
Owen G. et al. [49]	+	-	++	-	++	++	-	++	++
Graziano et al. (2008) [42]	+	-	++	-	++	++	-	++	++
Wei et al. (2020) [50]	+	-	++	-	++	+	-	++	++

## Data Availability

No new data were created or analyzed in this study. Data sharing is not applicable to this article.

## Abbreviations

The following abbreviations are used in this manuscript:

3D	Three-dimensional
ALP	Alkaline phosphatase
BMP-2	Bone morphogenetic protein-2
CCK-8	Cell Counting Kit-8
DOI	Digital Object Identifier
DPSC(s)	Dental pulp stem cell(s)
ECM	Extracellular matrix
ePTFE	Expanded polytetrafluoroethylene
FAK	Focal adhesion kinase
FN/CDHs (rFN/CDHs)	Recombinant fibronectin/cadherin fusion peptide
GBR	Guided bone regeneration
HA	Hydroxyapatite
HBDC(s)	Human bone-derived cell(s)
HGF(s)	Human gingival fibroblast(s)
hAMSC(s)	Human amnion mesenchymal stem cell(s)
hMSC(s)	Human mesenchymal stem cell(s)
hPDLSC(s)	Human periodontal ligament stem cell(s)
nHA	Nano-hydroxyapatite
OCN	Osteocalcin
OHAT	Office of Health Assessment and Translation
OSF	Open Science Framework
PDGF	Platelet-derived growth factor
PECVD	Plasma-enhanced chemical vapor deposition
PICO	Population, Intervention, Comparison, Outcome
PLA	Poly(lactic acid) / Polylactic acid
PLGA	Poly(lactic-co-glycolic acid)
PLLA	Poly(L-lactic acid)
PRISMA	Preferred Reporting Items for Systematic Reviews and Meta-Analyses
RoB	Risk of bias
RUNX2	Runt-related transcription factor 2
SiO_2_	Silicon dioxide (silica)
TCP	Tricalcium phosphate
TCPS	Tissue culture polystyrene
TiO_2_	Titanium dioxide
TGF-β1	Transforming growth factor beta 1
α5β1	Integrin alpha5 beta1
